# The relative contribution of target-site mutations in complex acaricide resistant phenotypes as assessed by marker assisted backcrossing in *Tetranychus urticae*

**DOI:** 10.1038/s41598-017-09054-y

**Published:** 2017-08-23

**Authors:** Maria Riga, Sabina Bajda, Christos Themistokleous, Stavrini Papadaki, Maria Palzewicz, Wannes Dermauw, John Vontas, Thomas Van Leeuwen

**Affiliations:** 1 Department of Biology, University of Crete, 70013 Heraklion Crete, Greece; 2 Institute of Molecular Biology & Biotechnology, Foundation for Research & Technology Hellas, 100 N. Plastira Street, 700 13 Heraklion Crete, Greece; 3 Institute for Biodiversity and Ecosystem Dynamics, University of Amsterdam, P.O. Box 9424, 1090 GE Amsterdam, The Netherlands; 4 Laboratory of Agrozoology, Department of Crop Protection, Faculty of Bioscience Engineering, Coupure Links 653, Ghent University, B-9000 Ghent, Belgium; 5 Laboratory of Pesticide Science, Department of Crop Science, Agricultural University of Athens, 75 Iera Odos Street, 11855 Athens, Greece

## Abstract

The mechanisms underlying insecticide and acaricide resistance in insects and mites are often complex, including additive effects of target-site insensitivity, increased metabolism and transport. The extent to which target-site resistance mutations contribute to the resistance phenotype is, however, not well studied. Here, we used marker-assisted backcrossing to create 30 congenic lines carrying nine mutations (alone, or in combination in a few cases) associated with resistance to avermectins, pyrethroids, mite growth inhibitors and mitochondrial complex III inhibitors (QoI) in a polyphagous arthropod pest, the spider mite *Tetranychus urticae*. Toxicity tests revealed that mutations in the voltage-gated sodium channel, chitin synthase 1 and cytochrome b confer high levels of resistance and, when fixed in a population, these mutations alone can result in field failure of acaricide treatment. In contrast, although we confirmed the implication of mutations in glutamate-gated chloride channels in abamectin and milbemectin insensitivity, these mutations do not lead to the high resistance levels that are often reported in abamectin resistant strains of *T. urticae*. Overall, this study functionally validates reported target-site resistance mutations in *T. urticae*, by uncoupling them from additional mechanisms, allowing to finally investigate the strength of the conferred phenotype *in vivo*.

## Introduction

Insecticide resistance is a major threat for the chemical control of insects and mites in public health and agriculture. At present, the Insecticide Resistance Action Committee (IRAC) distinguishes between at least fifty-five different chemical classes and more than twenty-five distinct mode of action (MoA) groups^1^. MoA diversity is of key importance for effective Insecticide Resistance Management (IRM). However, the costs involved in the discovery, development and marketing of chemicals with new properties, increased immensely and slow down the development of compounds with new MoA. In addition, concerns about the environment and human health, integrated in new regulations, demand molecules with better selectivity^2^. To preserve the utility and diversity of available and newly developed insecticides/acaricides, it is of utmost importance to understand the resistance mechanisms against these compounds^1^ and develop diagnostic tools that support monitoring activities and resistance management.

A number of mechanisms have been shown to underlie insecticide resistance, most often quantitative or qualitative changes in major detoxification enzymes and transporters (pharmacokinetic mechanisms) and/or target-site mutations (pharmacodynamic mechanisms)^3–5^. When resistance is caused by a combination of factors (polygenic resistance), the overall resistance levels may be the sum of contribution of each individual factor^6, 7^ but synergistic or antagonistic interactions between resistance loci also occur^8–10^. The relative contribution of each individual resistance locus to complex insecticide/acaricide resistance phenotypes has only been sporadically investigated^11^. In particular, the relative importance and strength of target-site mutations is often hard to assess by merely associating a phenotype with mutation frequency in field populations, where prolonged selection may have led to the accumulation of additional resistance mechanisms. Furthermore, the majority of studies that look into epistatic interactions and/or resistance levels confirmed by a single genetic factor, are sometimes difficult to interpret if resistance alleles are not investigated in a common genetic background^9, 12–15^. Therefore, analysis of a resistance trait requires the studied strains to be identical, except for its causal gene^16, 17^. Functional validation of resistance mutations has been reported after recombinant expression. Inhibitor-protein interactions are then quantified via enzymatic reactions or ligand binding assays such as voltage-clamp electrophysiology. Although they provide strong evidence of the effect of a mutation on the affinity for the compound to the target-site, they are less suitable to assess the relative phenotypic consequences *in vivo* ^18, 19^. A more precise way to determine the effect of a mutation *in vivo* is to introduce it in a defined susceptible genetic background, by utilizing genome editing techniques, such as CRISPR-Cas9^20, 21^, in species where this approach is applicable. In species where genome editing tools are not yet available, a more feasible alternative is to repeatedly backcross resistant individuals with susceptible ones^16, 22, 23^. Marker-assisted backcrossing provides a straight-forward and relatively precise method to untangle a mutation of interest from other mechanisms that might have been co-selected. The impact of a modifier or interactions between modifiers can be then analyzed by comparing the genetically identical strains that differ only in a small region on the chromosome, which harbors the resistant locus of interest^24, 25^.

The two-spotted spider mite, *Tetranychus urticae* (Chelicerata: Acari: Acariformes) is an important agricultural pest, that thrives on more than a 1,000 plant species^26, 27^. Its short life cycle, high fecundity and haplo-diploid system facilitates a rapid evolution of acaricide resistance. Today, *T. urticae* has developed resistance to more than 90 different chemical compounds, including major groups of currently used acaricides^1, 28, 29^. In *T. urticae* and other related spider mites, very high resistance ratios (RRs) have been reported for a number of compounds (RR > 10,000)^28, 30^ with cases of cross-resistance to newly introduced acaricides, for example, Khalighi, *et al*.^31^. Several target-site mutations have been uncovered and were associated with acaricide resistance in populations of *T. urticae*, recently summarized in Van Leeuwen and Dermauw^4^. These include mutations leading to amino acid substitutions in acetylcholinesterase (*AChE*) (G119S, A201S, T280A, G328A and F331W) that are associated with resistance to organophosphates and carbamates^32^. The L1024V and A1215D + F1538I substitutions in the voltage-gated sodium channel (VGSC) have been linked to resistance to Type I (absence of α-cyano group) and Type II (presence of α-cyano group) pyrethroids^33, 34^. Six orthologous glutamate–gated chloride channel (GluCl) genes have been reported in spider mites and the substitutions G314D and G326E in GluCl1 and GluCl3, respectively, were associated with resistance to abamectin^35, 36^. The G126S, I136T, S141F, D161G, P262T substitutions (in different combinations) identified in the cytochrome b (cytb) cause strong bifenazate resistance (Mitochondrial Qo inhibitors: QoI)^37^. A substitution I1017F in the chitin synthase 1 gene (*CHS1*) has been linked with high levels of resistance to mite growth inhibitors, etoxazole, clofentezine and hexythiazox^38, 39^. Most recently, an H92R substitution in the PSST subunit of the Mitochondrial Respiratory Complex I, has been associated with resistance to pyridaben, tebufenpyrad and fenpyroximate (Mitochondrial Electron Transport Inhibitors, site I, METI-I)^25^. As resistance in spider mites often has a polygenic basis, the relative contribution of target-site resistance to the overall resistance levels is currently unknown. One notable exception for *T. urticae* is the H92R mutation in the PSST subunit, which was introduced into a susceptible background by repeated backcrossing and shown to confer moderate levels of METI resistance^25^.

In this study, we investigated the relative contribution of nine known target-site mutations conferring resistance to abamectin, pyrethroids, bifenazate and mite growth inhibitors. We adopted the method of Bajda, *et al*.^25^ and succeeded in generating 30 congenic resistant and susceptible lines of *T. urticae*. When a combination of mutations in homologous genes was reported, the phenotypic levels of resistance were examined for both the single mutations, as well as their combination.

## Materials and Methods

### Acaricides

Acaricides used in this study were commercial formulations of bifenazate (Floramite, 240 g l^−1^ SC) and acequinocyl (Cantack 164 g l^−1^ SC), etoxazole (Borneo, 120 g l^−1^ SC), hexythiazox (Nissorun, 250 g l^−1^ SC) and clofentezine (Apollo, 500 g l^−1^ SC), abamectin (Vertimec 18 g l^−1^ EC), milbemectin (Milbeknock 10 g l^−1^ EC), bifenthrin (Talstar 100 g l^−1^ EC), fluvalinate (Mavrik 240 g l^−1^ EW) and analytical grade fenpropathrin (Sigma Aldrich).

### Spider mite strains

The susceptible Wasatch strain is an inbred line, originally collected from tomato in a greenhouse near Salt Lake City, Utah, USA. The pyrethroid susceptible strain KOP8 is an inbred line derived from the Houten strain^40^. Wasatch does not contain any of the so far described mutations. KOP8 harbors the A1215D substitution, potentially associated with pyrethroid resistance. The GH strain carries the L1024V genotype (*Musca domestica* numbering) of the VGSC gene and was collected from greenhouse grown maize in Utah USA. The TuSB9 strain carrying the A1215D and F1538I mutations (*Musca domestica* numbering) in VGSC was previously described^33^. The MAR-AB strain carrying the G314D and G326E substitutions (*Tetranychus urticae* numbering) in GluCl1 and GluCl3, respectively, was previously described in Dermauw, *et al*.^35^. Strains with mutations associated with bifenazate resistance, HOL3 (cytb, P262T - *Tetranychus urticae* numbering) and BR-VL (cytb, G126S and S141F – *Tetranychus urticae* numbering) were described in Van Leeuwen, *et al*.^37^ and Van Leeuwen, *et al*.^41^ respectively. The EtoxR strain carrying the I1017F mutation (*Tetranychus urticae* numbering) in the chitin synthase (CHS1) gene was previously described^38^. An overview of strains is presented in Table 1. All *T. urticae* strains were maintained on 3-week old potted kidney bean plants (*Phaseolus vulgaris L*.) in a climatically controlled room or incubator at 25 ± 1 °C, 60% relative humidity, and 16:8 light: dark photoperiod.

**Table 1:** Summary of crosses performed to create congenic *T. urticae* lines.

strain	resistant to*	target-sitemutation	crossed to	backcrossed lines	
MAR-AB	abamectin (6)^3^	GluCl1 (G314D)	Wasatch	GluCl1_C, GluCl1_R1, R2, R3	GluCl1 + 3_C, GluCl1 + 3_R1, R2, R3
MAR-AB	abamectin (6)^3^	GluCl3 (G326E)	Wasatch	GluCl3_C, GluCl3_R1, R2, R3	
GH	pyrethroids (3 A)^3^	VGSC (L1024V)	Wasatch	VGSC _C1, VGSC _R1, R2, R3	
TuSB9	pyrethroids (3 A)^3^	VGSC (F1538I + A1215D)	KOP8	VGSC_C2, VGSC_R4, R5	
HOL3	bifenazate (20 A)^3^	cytochrome b (P262T)	Wasatch	cytb_R1, R2, R3	
BR-VL	bifenazate (20 A)^3^ acequinocyl (20B)^3^	cytochrome b (G126S + S141F)	Wasatch	cytb_R4, R5	
EtoxR	mite growth inhibitors (10)^1,2^	chitin synthase (I1017F)	Wasatch	CHS1_C, CHS1_R1, R2, R3	

VGSC mutations were numbered according to *Musca domestica* numbering, whereas GluCl1, GluCl3, cytochrome b and chitin synthase substitutions according to *Tetranychus urticae* numbering. *IRAC mode of action group number is shown between brackets. Superscript numbers indicate which mite stage was used in the toxicity assay (1: larval toxicity assay, 2: egg toxicity assay, 3: adult toxicity assay). Refer to section Toxicity bioassays for more details.

### Backcrossing experiments

**Figure 1:**
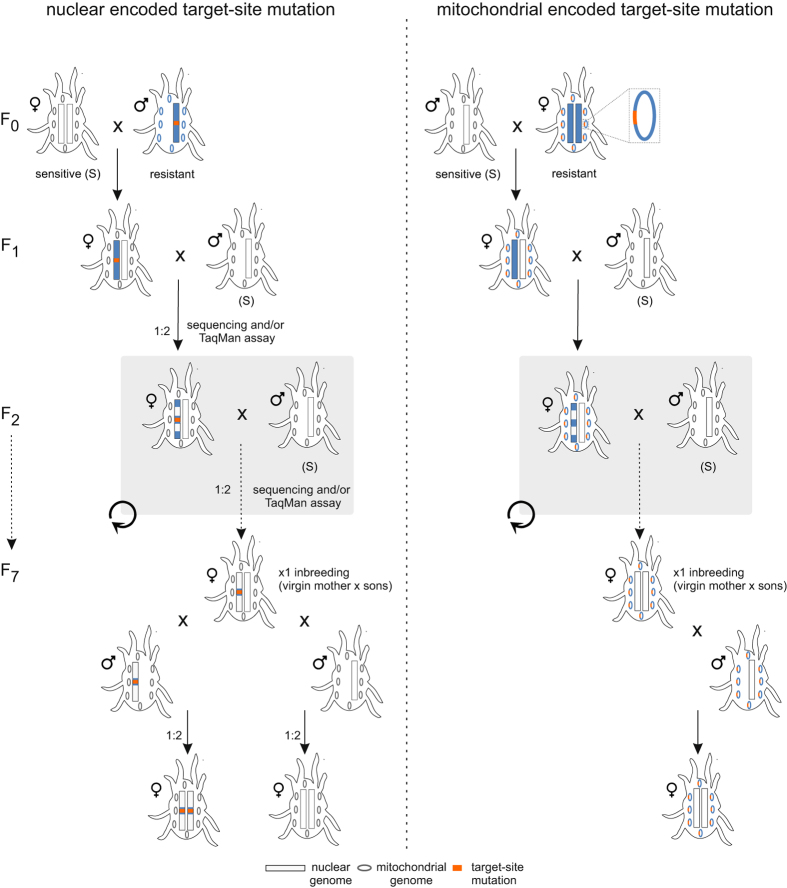
Schematic diagram of marker-assisted backcrossing of nuclear and mitochondrial encoded resistance mutations. The susceptible genotype is depicted by white-colored chromosomes (rectangles) and mitochondria (ovals), while those of the resistant genotype are depicted in blue. An orange color indicates whether the resistance mutation is either nuclear or mitochondrial encoded.

To assess the relative resistance levels associated with mutations, we used a marker assisted backcrossing approach to produce near-isogenic sister lines (Fig. 1 and Table 1). The crossing procedure was previously outlined in Bajda, *et al*.^25^. In short, a haploid male of the resistant strain was crossed with a virgin female of the susceptible strain. The resulting heterozygous virgin females were backcrossed to susceptible males and heterozygote genotypes were identified by a TaqMan molecular assay or PCR and sequencing as it is described in section Genotyping. This process was repeated for six to nine generations. In the last generation, a cross was carried out between the backcrossed heterozygous virgin females and their first born sons representing either a susceptible (absence of mutation) or the resistant (presence of mutation) genotype. This finally resulted in congenic homozygous lines for the mutation and the wild type allele. The final crosses were performed as follows (see Table 1): For the mutations in GluCls, G314D in GluCl1 and G326E in GluCl3, MAR-AB males were crossed with Wasatch virgin females in order to separate the mutations in different lines, as they are inherited independently^35^, after which they were introgressed separately: ♀ 314D/314 G x ♂ 314D or ♂ 314 G to generate GluCl1_R1-R3 and GluCl1_C, ♀326E /326 G x ♂ 326E or ♂326 G to produce homozygous congenic GluCl3_R1-R3 and GluCl3_C respectively. Mutations were later joined in a single line by dedicated crosses as follows: ♀GluCl1_R1 x ♂GluCl3_R1, ♀GluCl1_R2 x ♂GluCl3_R2, ♀GluCl1_R3 x ♂GluCl3_R3 and ♀GluCl1_C x ♂GluCl3_C to produce GluCl1 + 3_R1,R2,R3 and C respectively. For the mutations in VGSC; the ♀1024 V/1024 L x ♂1024 V or ♂1024 L were crossed to obtain homozygous congenic lines VGSC_R1-R3 and VGSC_C1 respectively, ♀1215D + 1538I/1215D + 1538 F x ♂1215D + 1538I or ♂1215D + 1538 F to obtain homozygous congenic VGSC_R4-R6 and VGSC_C2 respectively. For the mutation in CHS1; ♀ 1017 F/1017I x ♂ 1017 F or ♂1017I were crossed to generate homozygous congenic CHS1_R1-R3 and CHS1_C, respectively.

For the mitochondrial mutations in cytb (HOL3 and BR-VL) that are inherited completely maternally, simple crosses between resistant females and susceptible males were performed for 7 generations, as to create a line with the nuclear genome of the susceptible parent (Wasatch), but bearing the mitochondrial haplotype of the resistant line (Fig. 1). Three crosses were set up to produce lines carrying P262T mutation in PEWY motif of cytb (cytb_R1-R3) and consequently three lines with G126S + S141F mutations in cd1 region of cytb (cytb_R4-R6).

### Single mite DNA extraction

In order to perform single mite genotyping for I1017F (EtoxR), P262T (HOL3) and G126S + S141F (BR-VL) individual *T. urticae* mites were homogenized in 20 μl STE buffer (100 mM NaCl, 10 mM Tris- HCl and 1 mM EDTA) with 1 mg ml^−1^ proteinase K (Sigma-Aldrich). Homogenate was incubated at 37 °C for 30 min followed proteinase K inactivation for 5 min at 95 °C. For G314D, G326E (MAR-AB) and F1538I, A1215D (TuSB9), L1024V (GH) single mite DNA was extracted following the CTAB method^42^. In short, individual mites were homogenized in 200 μl of extraction buffer (2% CTAB, 1.4 M NaCl, 0.2% β-mercaptoethanol, 20 mM EDTA, 100 mM Tris – HCl, pH:8.0) and incubated at 65 °C for 15 min. Equal volume of chloroform: isoamylalcohol (24:1) was used in order to remove proteins. The DNA was precipitated by isopropanol and washed with 75% ethanol. The pellet was air-dried and resuspended in 20 μl DEPC treated water.

### Genotyping

Single mite genotyping was performed with standard PCR and sequencing (mutations I1017F, P262T, G126S + S141F and L1024V) and/or TaqMan method^43^ (mutations F1538I, G314D and G326E). PCRs were conducted in 50 μl final volume with 10 μl 5x Phusion HF Buffer, 0.2 mM of each dNTP, 0.5 μM each primer, 1 μl template, 0,5 μl polymerase with cycling conditions; 30 s at 98 °C followed by 35 cycles 5 s at 98 °C, 10 s at 55 °C, 15 s at 72 °C and 5 min of final extension. Reactions were performed in Bio-Rad T100™ Thermal Cycler. PCR products were purified with E.Z.N.A.® Cycle Pure Kit DNA purification kit (OMEGA bio-tek) and sequenced at Macrogen sequencing facility (Amsterdam). Sequencing data were analyzed using BioEdit 7.0.1 software^44^. Primers used for the PCR reactions and sequencing are listed in Supplementary Table S1.

TaqMan assay was performed as previously described^43^. In short, all assays were carried out in 15 μl total volume containing 2 μl of genomic DNA, 7.5 μl TaqMan Universal PCR Master Mix, 0.8 μM of each primer and 0.2 μM of each probe. Samples were run on CFX Connect, Real-Time PCR Detection System (Biorad) using the temperature cycling conditions of: 10 min at 95 °C followed by 40 cycles of 92 °C for 15 sec and 60 °C for 1 min. The increase in VIC and FAM reporter dyes, representing individuals with the resistant and susceptible alleles respectively, was monitored in real time using the CFX Manager software. Positive and negative template controls were included in each run to aid genotype scoring. Primers and probes used for the TaqMan assay are listed in Supplementary Table S1.

### Toxicity bioassays

To assess the toxic effects of etoxazole and hexythiazox, larval bioassays (1) were performed as previously described by Van Pottelberge, *et al*.^45^. For the ovicide clofentezine, bioassays were performed on eggs (2) instead of larvae. Approximately fifty females were allowed to lay eggs for 5 hours on the upper side of 9 cm^2^ square-cut kidney bean leaf discs on wet cotton wool. For adulticidal bioassays (3)^46^, 20–30 young adult female mites were transferred to arenas, prepared as described above. Plates were sprayed with 1 ml of spray fluid at 1 bar pressure with a Potter Spray Tower (Burkard Scientific, UK) to obtain a homogenous spray film (2 mg deposit/cm^2^). Experiments were then placed in a climatically controlled room at 25 ± 0.5 °C, 60% RH and 16/8 h (light/dark) photoperiod. Three to four replicates of at least five serial dilutions of each acaricide and a control (deionized water or 1:100 dilution of the mixture of N, N-dimethylformamide and emulsifier W, depending on the acaricide used) were tested. Fenpropathrin was of technical grade and formulated in 3:1 v/v mixture of N, N-dimethylformamide and emusulfier W and subsequently diluted in deionized water as previously described^47^. Mortality was assessed after 24 h for bifenazate and acequinocyl and 48 h for all other acaricides. Mites treated with growth inhibitors, were considered as unaffected, if at the time of scoring displayed the same developmental stage as water treated control. Adult mites were scored as being alive if they could walk twice the distance of their body size after being prodded with a camel’s hair brush^48^. All mortalities obtained for control treatment were lower than 10%. LC_50_ values, slopes, RRs and 95% confidence limits were calculated by probit analysis (POLO, LeOra Software, Berkeley, USA)^49^. In case 5,000 mg l^−1^ did not cause 50% mortality, no further attempts were made to determine LC_50_s and RR was calculated by dividing 5,000 mg l^−1^ by the LC_50_ of susceptible strain. The effect of the treatment on the susceptible parent and the experimental line was considered significantly different if the hypothesis of equality of slopes and intercepts was rejected (p value = 0.05)^50^. If a regression line - illustrating dose response - could not be derived (LC_50_ of the experimental line was found to be higher than 5,000 mg l^−1^), the effect of treatment was considered different when the LC_90_ of the susceptible control was lower than 5,000 mg l^−1^.

### Data availability

All data generated or analyzed during this study are included in this manuscript (and its Supplementary Information Files).

## Results

### Establishment of congenic lines

The initial crosses between parental resistant and susceptible strains are outlined in Table 1. Briefly, the susceptible strain Wasatch, which does not carry any of the mutations studied here, was used for the most of the backcrossing experiments (Table 1). To study the mutations in GluCl1 (G314D) and GluCl3 (G326E) associated with abamectin resistance, virgin females of Wasatch were crossed with males of the abamectin resistant strain MAR-AB carrying both GluCl mutations. Similarly, for the L1024V mutation associated with pyrethroid resistance, Wasatch virgin females were crossed with males of the pyrethroid resistant strain GH that carries L1024V. The effect of A1215D + F1538I mutations in pyrethroid resistance was examined through crossing males of TuSB9 with females of the parental susceptible strain KOP8 (carrying the A1215D only). To study the effect of mutations in the mitochondrial encoded cytb (P262T and G126S + S141F) that confer resistance to bifenazate, virgin females of bifenazate resistant strains HOL3 (P262T) and BR-VL (G126S + S141F), were crossed to males of Wasatch. Last, to introduce the mutation I1017F associated with resistance to mite growth inhibitors, virgin females of Wasatch were crossed with EtoxR males (Table 1).

For the nuclear encoded mutations, the final cross between heterozygous backcrossed females and their sons resulted in congenic homozygous lines with either the mutation fixed or absent (Fig. 1, Table 1, see Backcrossing experiments paragraph for outline of experimental setup). Since mutations in GluCl1 and GluCl3 are not genetically linked^35^, the impact of each mutation could be assessed separately. Once homozygous backcrossed lines carrying a mutation either in GluCl1 (GluCl1_R1-R3) or in GluCl3 (GluCl3_R1-R3) and their respective congenic control lines (GluCl1_C and GluCl3_C) were generated, the mutations were joined again by dedicated crosses, giving rise to GluCl1 + 3_R1-R3. The susceptible control GluCl1 + 3_C was obtained with the cross GluCl1_C x GluCl3_C. One replicate with genotype A1215D + F1538I (pyrethroid resistance mutations) and one with genotype G126S + S141F (bifenazate resistance mutations) were lost during backcrossing and only two biological replicates VGSC_R4, R5 and cytb_R4 and R5 could be analyzed for each genotype.

### Toxicity assays

#### Parental strains

Abamectin and milbemectin were tested against the parental susceptible strain, Wasatch and the resistant strain, MAR-AB (G314D + G326E), with the latter one exhibiting high resistance levels to abamectin (1,354.9 fold) and moderate resistance to milbemectin (71.7 fold) in comparison to Wasatch (Supplementary Table S2).

The parental susceptible strains, KOP8, which carries only the A1215D VGSC substitution, and Wasatch showed high susceptibility to bifenthrin, fluvalinate and fenpropathrin whereas the GH (L1024V) and TuSB9 (A1215D + F1538I) resistant strains were highly resistant to the aforementioned pyrethroids (Table 2).

**Table 2:** Toxicity of pyrethroids (bifenthrin, fluvalinate and fenpropathrin) to adult females of backcrossed lines VGSC_C1, VGSC_R1-R3 (L1024V genotype), VGSC_C2, VGSC_R4,5 (F1538I + A1215D genotype) and their parental strains (Wasatch, GH, KOP8, TuSB9).

Compound	Strain	Genotype	N^a^	LC_50_ mg l^−1^ (95% CI)	Slope ( ± SE)	χ^2^ (df)	RR (95% CI)^b^
bifenthrin	Wasatch	L1024	404	3.8 (2.1; 4.7)	3.9 (±0.8)	17 (13)	—
	GH	L1024V	443	1,031.0 (721.7; 1,406.8)a	1.5 (±0.1)	14 (13)	271.8 (185.3; 398.8)
	KOP8	A1215D + F1538	354	4.1 (3.0; 4.8)	3.2 (±0.6)	8 (16)	—
	TuSB9	A1215D + F1538I	517	1,715.8 (696.5; 2,474.8)a	2.3 (±0.4)	24 (16)	423.5 (272.4; 658.4)
	VGSC_C1	L1024	382	5.09 (3.4; 6.2)a	4.9 (±0.8)	26 (13)	1.3 (1.0; 1.8)
	VGSC_C2	A1215D + F1538	436	4.6 (3.3; 5.5)	4.8 (±0.8)	29 (16)	1.1 (0.9; 1.5)
	VGSC_R1	L1024V	670	353.3 (277.1; 410.3)a	3.7 (±0.6)	20 (19)	93.2 (69.1; 125.7)
	VGSC_R2	L1024V	560	328.2 (260.7; 390.5)a	3.0 (±0.5)	13 (18)	86.5 (63.1; 118.8)
	VGSC_R3	L1024V	427	405.4 (329.8; 466.5)a	3.8 (±0.7)	13 (13)	106.9 (79.4; 143.9)
	VGSC_R4	A1215D + F1538I	554	508.9 (261.6; 670.8)a	2.6 (±0.6)	16 (12)	125.6 (87.5; 180.3)
	VGSC_R5	A1215D + F1538I	435	538.8 (380.6; 670.2)a	3.6 (±0.5)	21 (12)	134.0 (100.4; 176.1)
fluvalinate	Wasatch	L1024	479	102.2 (82.7; 118.5)	3.9 (±0.6)	18 (17)	—
	GH	L1024V	118	>5,000a	—		>45
	KOP8	A1215D + F1538	294	92.4 (67.3; 117.5)	4.7 (±1.1)	15 (11)	—
	TuSB9	A1215D + F1538I	186	>5,000a	—	—	>50
	VGSC_C1	L1024	436	83.0 (63.2; 98.5)	3.7 (±0.6)	16 (15)	0.8 (0.6; 1.0)
	VGSC_C2	A1215D + F1538	508	87.0 (69.3; 102.4)	3.7 (±0.5)	19 (15)	0.9 (0.8; 1.2)
	VGSC_R1	L1024V	188	>5,000a	—	—	>45
	VGSC_R2	L1024V	180	>5,000a	—	—	>45
	VGSC_R3	L1024V	213	>5,000a	—	—	>45
	VGSC_R4	A1215D + F1538I	194	>5,000a	—	—	>50
	VGSC_R5	A1215D + F1538I	161	>5,000a	—	—	>50
fenpropathrin	Wasatch	L1024	360	21.3 (15.8; 26.9)	3.1 (±0.5)	23 (19)	—
	GH	L1024V	97	>5,000a	—	—	>230
	KOP8	A1215D + F1538	297	13.7 (11.0; 16.9)	2.8 (±0.5)	8 (15)	—
	TuSB9	A1215D + F1538I	182	>5,000a	—	—	>360
	VGSC_C1	L1024	476	35.2 (26.2; 44.2)a	2.1 (±0.3)	5 (16)	1.7 (1.2; 2.3)
	VGSC_C2	A1215D + F1538	396	21.5 (15.9; 26.8)a	3.5 (±0.5)	15 (19)	1.6 (1.1; 2.2)
	VGSC_R1	L1024V	153	>5,000a	—	—	>230
	VGSC_R2	L1024V	155	>5,000a	—	—	>230
	VGSC_R3	L1024V	180	>5,000a	—	—	>230
	VGSC_R4	A1215D + F1538I	171	>5,000a	—	—	>360
	VGSC_R5	A1215D + F1538I	156	>5,000a	—	—	>360

^a^Number of mites used in toxicity tests. ^b^RR compared to Wasatch in case of GH, VGSC_C1 and VGSC_R1-3 or KOP8 in case of TuSB9, VGSC_C2 and VGSC_R4,5 lines. a: Treatment effect was significantly different when compared to Wasatch or KOP8.

**Figure 2:**
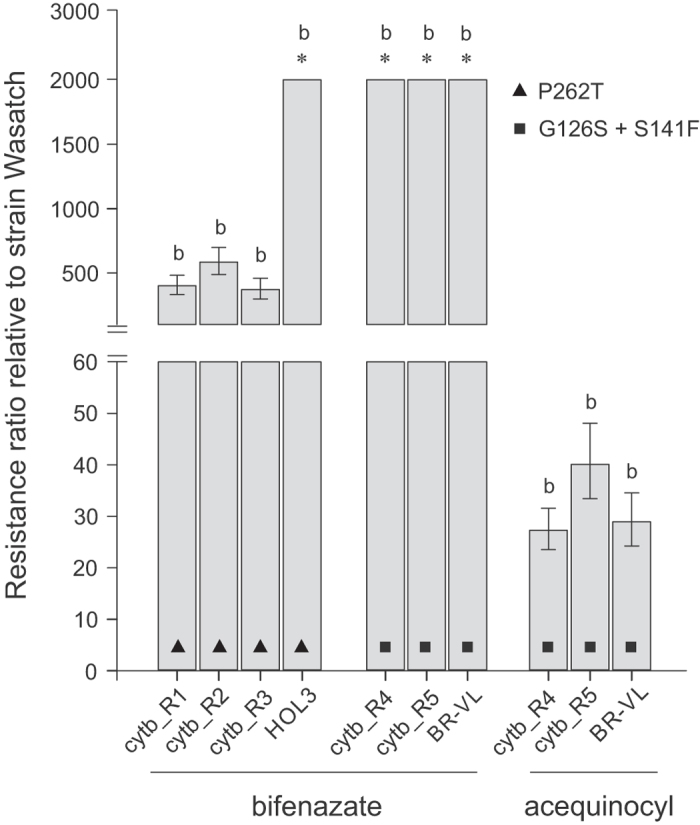
Susceptibility of backcrossed *T. urticae* lines cytb_R1-R3 (P262T) and cytb_R4, R5 (G126S + S141F) and the resistant parental strains HOL3 and BR-VL to QoI acaricides bifenazate and acequinocyl. The RRs were calculated as the LC_50_ values of the backcrossed lines divided by the LC_50_ of the parental susceptible strain Wasatch. Stars indicate strains for which the LC_50_ value exceeded 5,000 mg l^−1^. Error bars represent 95% confidence limits calculated by probit analysis. Letters above bars indicate lines where acaricide treatment had statistically the same (**a**) or different (**b**) effect comparing to Wasatch (PoloPlus LeOra Software)^49^.

The Wasatch strain and the parental resistant strains HOL3 (P262T) and BR-VL (G126S + S141F) were tested against bifenazate. The resistant strains exceeded 2,000 fold of resistance to bifenazate. Additionally, the parental susceptible strain and BR-VL were treated with acequinocyl, with the latter one showing moderate levels of resistance (RR of 28.9 fold) (Fig. 2, Supplementary Table S3).

**Figure 3:**
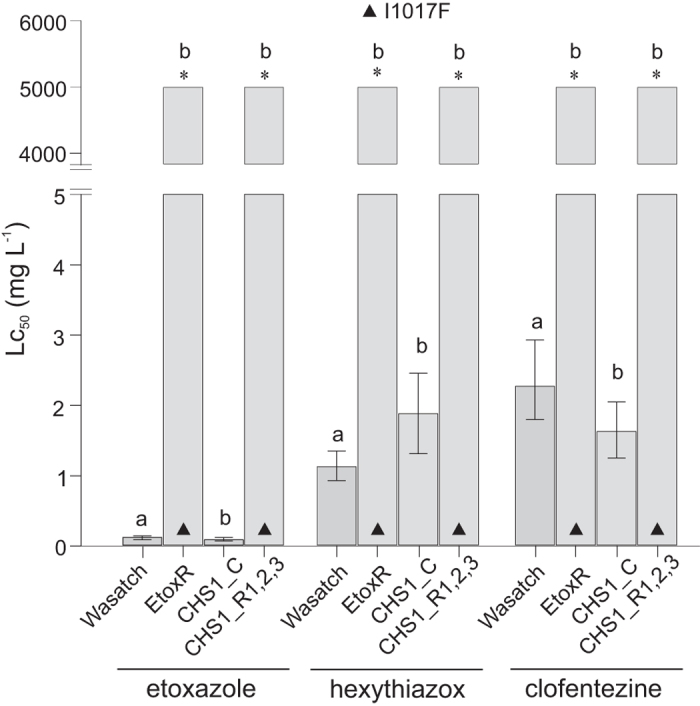
Susceptibility of backcrossed *T. urticae* lines CHS1_R1-R3 (I1017F) and CHS1_C and their susceptible and resistant parental strains, to mite growth inhibitors etoxazole (most left), hexythiazox (middle) and clofentezine (most right). Bars represent the acaricide concentration at which 50% of the individuals are affected. Error bars represent the 95% confidence limit calculated by probit analysis. As LC_50_ values exceeded 5,000 mg l^−1^ for all CHS1_R1, R2 and R3 for each mite growth inhibitor tested, only one bar depicts LC_50_s. Stars indicate lines for which, LC_50_ value exceeded 5,000 mg l^−1^. Letters above bars indicate lines where acaricide treatment had statistically the same (**a**) or different (**b**) effect comparing to Wasatch (PoloPlus LeOra Software)^49^.

Etoxazole, clofentezine and hexythiazox were tested against the EtoxR strain (I1017F) and the susceptible strain Wasatch. The EtoxR strain showed extremely high levels of insensitivity to all three compounds used, with RR values exceeding 40,000 for etoxazole, 4,000 for hexythiazox and 2,000 for clofentezine (Supplementary Table S4, Fig. 3).

#### Backcrossed strains

**Figure 4:**
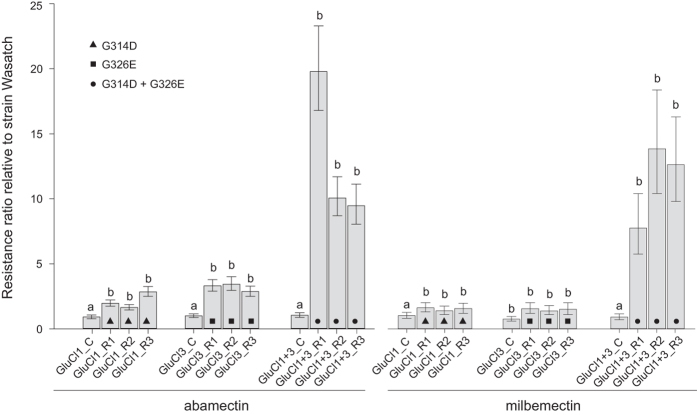
Susceptibility levels of backcrossed *T. urticae* lines GluCl1_R1-R3 (G314D), GluCl1_C, GluCl3_R1-R3 (G326E), GluCl3_C, GluCl1 + 3_R1-R3 (G314D + G326E), GluCl1 + 3_C to abamectin and milbemectin. The RRs were calculated as the LC_50_ values of the backcrossed lines divided by the LC_50_ of the parental susceptible strain Wasatch. Error bars represent the 95% confidence limit calculated by probit analysis. Letters above bars indicate lines where acaricide treatment had statistically the same (**a**) or different (**b**) effect comparing to Wasatch (PoloPlus LeOra Software)^49^.

Abamectin and Milbemectin. The introgressed strains carrying resistance mutation in only one of the GluCls (either GluCl1 or GluCl3) showed minor resistance to abamectin and milbemectin with RR values up to 3.3 and 1.6 folds, respectively (Fig. 4, Supplementary Table S2). However, when mutations were joined by dedicated crosses, individuals carrying both mutations (GluCl1 + 3_R1-3 congenic lines) showed higher resistance levels to both compounds. The RR values obtained for abamectin and milbemectin were up to 19.8 and 13.7 fold, respectively (Fig. 4, Supplementary Table S2).

Pyrethroids. The backcrossed strains VGSC_R1-3 and VGSC_R4,5 exhibited high levels of resistance to all pyrethroids used in this study (bifenthrin, fluvalinate and fenpropathrin), with RR values being greater than 200 fold in some cases. In contrast, the backcrossed susceptible lines VGSC_C1 and VGSC_C2 were susceptible to all three compounds (Table 2).

Mitochondrial QoI. The backcrossed strains cytb_R1, R2 and R3 carrying the P262T mutation in cytb sequence showed high resistance levels to bifenazate (Fig. 2, Supplementary Table S3). Interestingly, the combination of cytb substitutions; G126S and S141F provided higher level of resistance to bifenazate compared to P262T, since RRs for both cytb_R4 and R5 were higher than 2,000 fold. The importance of G126S and S141F in the observed levels of cross-resistance to acequinocyl in BR-VL was confirmed (Fig. 2, Supplementary Table S3).

Mite growth inhibitors. The backcrossed strains homozygous for I1017F mutation displayed extreme levels of resistance to all three mite growth inhibitors tested (Fig. 3, Supplementary Table S4). RRs estimated for etoxazole, hexythiazox and clofentezine exceeded 40,000, 4,000 and 2,000 fold, respectively (Fig. 3, Supplementary Table S4). In contrast, the backcrossed control line was highly susceptible to the aforementioned compounds (Fig. 3, Supplementary Table S4).

## Discussion

Field collected *T. urticae* strains often exhibit very high levels of resistance to multiple acaricides used for their control. Due to the identification of acaricide target-site sequences^29, 51^ and implementation of recently developed genetic mapping tools^4, 25, 38^, a number of mutations has been uncovered in the target-site of frequently used acaricides. However, to what extent these mutations determine the resistant phenotype is mostly unknown. Resistant field strains investigated so far, typically display a broad altered transcriptional response with the putative involvement of many detoxifying enzymes and transporters that might affect acaricide toxicity^52–54^. Crossing experiments have revealed that a complex genetic make-up typically underlies resistance, implying the additive effect of multiple mechanisms^35, 55, 56^. Moreover, the extent by which resistant alleles confer resistance can also vary according to the genetic background in which they are expressed^57, 58^.

Several studies have used congenic backcrossed lines to assess insecticide related fitness cost/advantage and pleiotropic effects^59–64^. By substituting phenotypic selection with molecular marker-assisted backcrossing, the potential accumulation of alleles with additive effect can be uncoupled^23^. Such a setup has been previously used to assess the effects of *Aedes aegypti* kdr mutations on pyrethroid resistance and its fitness cost^24^ and recently, to investigate resistance levels to METI-I acaricides caused by a mutation in the PSST subunit of complex I in *T. urticae* ^25^.

Here, we analyzed the relative phenotypic contribution of target-site resistance mutations, previously uncovered in highly resistant *T. urticae* field populations. We adopted a marker-assisted backcrossing procedure described in Bajda, *et al*.^25^ to untangle the target-site resistance loci from potential complex additive genetic mechanisms. Although in this study we cannot exclude a possible effect of closely linked loci^65^, previous research involving resistance gene mapping by means of bulk segregant analysis, revealed a high recombination rate in *T. urticae* ^38, 39^ which makes us believe that the procedure performed here, resulted in near-isogenic lines.

**Figure 5:**
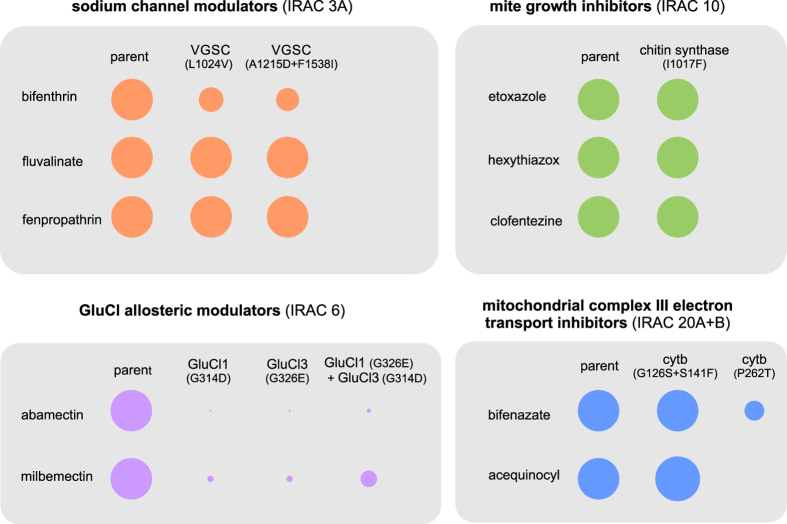
Schematic representation of the relative contribution of target site resistance mutations in overall resistance levels to acaricides belonging to different mode of action group﻿s. The size of the circle shape reflects the observed levels of resistance (RR vs susceptible parent strain). Only comparisons between the backcrossed lines versus its resistant parent are drawn to scale (Table 1).

Both abamectin and milbemectin resistance has been reported frequently in spider mite populations worldwide^48, 66, 67^ exhibiting >1000 fold resistance in some cases^35^. These molecules target both GluCls and GABA gated chloride channels (GABACl), although GluCls are considered the main target^68, 69^. In contrast to insects with a single copy, the genome of *T. urticae* harbors six orthologous GluCl genes^35^. Two non-synonymous mutations have been associated with resistance to abamectin, the G314D in GluCl1 and G326E in GluCl3^35, 36^. When G314D and G326E were introgressed separately, only low levels of resistance remained. However, when both mutations were joined by dedicated crosses, resistance levels increased to 10–20 fold (henceforth, for the schematic visualization of a relative contribution of the mutations in resistance levels, please consult Fig. 5). These resistance levels are comparable with a previous study, where an abamectin resistant strain homozygous for both GluCl mutations was investigated. Resistance levels in that strain reached only 20-fold^36, 70^, suggesting that target-site mutations were the only factor contributing to resistance. A possible explanation for the relatively low resistance levels conferred by the combination of two GluCl mutations may lie in the number of genes involved in channel assembly. Glutamate-gated chloride channels typically consist of five subunits, which in *T. urticae* can be encoded by 5 different GluCl genes. Hence, if the channel consists of a combination of subunits carrying the resistance associated substitution (GluCl1 and/or GluCl3) and a GluCl2 subunit (GluCl2 does not carry a resistance associated substitution, while GluCl4 and GluCl5 naturally carry substitutions that interfere with abamectin binding see Dermauw, *et al*.^35^), abamectin binding might still be possible. In addition, we cannot exclude the possibility of heteromeric channel assembly, consisting of GluCls and GABACl^18, 19^. In such case, the existence of mutations in GluCl1 and GluCl3 alone would also not be capable to fully prevent channel blocking. Consequently, our results also reconfirm the importance of additional mechanisms in abamectin resistance^69, 71, 72^. Studies with synergists and biochemical tests have previously implied the involvement of detoxification enzymes in resistance in many field collected strains worldwide^72–74^. For instance, a P450 (CYP392A16) was reported to be overexpressed in abamectin resistant strains and detoxifies abamectin rapidly^71^. Therefore very high abamectin resistance levels in the MAR-AB strain (Supplementary Table S2) may be attributed to a joint action of P450 detoxification and decreased sensitivity of the target-site, potentially even acting synergistically.

Milbemectin belongs to the same insecticidal class as abamectin and acts on the same target-site. Whether cross-resistance may occur between both compounds is therefore of crucial importance, and still a matter of debate. Here, we show that the combination of both GluCl mutations confers resistance levels of about 10-fold, indicating potential cross-resistance risks between milbemectin and abamectin, as has been previously suggested^48, 67^.

Pyrethroid resistance has been documented globally in *T. urticae* with resistance levels exceeding 10,000 folds in some cases^75, 76^. Unlike most other arthropods, spider mites have mutations in unique positions on VGSC^33, 34, 77^, instead of the known kdr (L1014F) and super-kdr (M918T) mutations (*Musca domestica* numbering). The super-kdr mutation has been identified only once in a *Tetranychus evansi* strain^78^. Three point mutations have been located in the sodium channel of spider mites, L1024V and F1538I in combination with A1215D^33, 34^. Backcrossing experiments indicated the major effect of both L1024V and A1215D + F1538I mutations in pyrethroid resistance (Fig. 5). Interestingly, the KOP8 strain has the A1215D mutation uncoupled from F1538I and is susceptible to all pyrethroids, indicating that the mutation alone has no effect on pyrethroid toxicity. So far, the mutation F1538I has been studied most thoroughly and its effect in resistance to pyrethroids has been confirmed by electrophysiological studies^79^. Here, we showed that both L1024V and A1215D + F1538I mutations confer high resistance levels to all pyrethroid compounds, irrespectively of their type, i.e. presence of α-cyano group and/or extended halogenated acidic moiety, suggesting that the sodium channel mutations can cause field failure of the pyrethroids.

QoI with acaricidal properties have been introduced for the control of mite infestations relatively recently^80, 81^. Nonetheless, high levels of resistance to bifenazate have already been reported in the field^37, 82^. Backcrossing revealed that the combination of cd1 helix mutations G126S and S141F in cytb confers high levels of bifenazate resistance. In contrast, the backcrossed lines carrying the P262T substitution, showed LC_50_ values of 960, 1,400.6 and 886.1 mg l^−1^, respectively, while the parental resistant HOL3 was resistant to bifenazate concentrations up to 5,000 mg l^−1^ (Fig. 5). The backcrossed lines with cd1 helix mutations, showed similar levels of resistance to acequinocyl compared to the parental strain, confirming that acequinocyl cross-resistance is completely maternally inherited, and thus linked with the mutation^82^. One of the possible explanations for the discrepancy between bifenazate resistance of the parental strain HOL3 and those of the backcrossed strains is the presence of additional resistance mechanisms. Indeed, although a strong correlation between the P262T frequency and bifenazate resistance has been reported, to what extent resistance is inherited maternally has only been described for acequinocyl^82^. This is in contrast to cd1 helix mutations, that have been shown to confer resistance levels that are completely maternally inherited^41^, suggesting that no additional mechanisms are involved or needed to attain very high resistance levels. Another explanation could be that the P262T substitution only confers high resistance levels in combination with specific nuclear encoded protein variants that co-evolved with mitochondrial encoded cytb mutations, and that uncoupling results in a loss of phenotype.

Resistance to clofentezine and hexythiazox has been frequently reported, and more recently, resistance against etoxazole has been also spreading^83–85^. Insensitivity to etoxazole is thought to be monogenic and recessive^84, 85^, which is in line with target-site resistance as the main mechanism. Screening for I1017F revealed that the mutation has been segregating in populations from different regions of the world for a long period, although etoxazole has been only recently used to control spider mites, especially in Europe^38^. This lead to the hypothesis that the mutation was selected by other molecules such as hexythiazox or clofentezine, which was later confirmed in a follow-up genetic mapping study^39, 86^. Here, we provide clear evidence that the I1017F substitution confers very high levels of resistance to etoxazole, hexythiazox and clofentezine (Fig. 5). Our results confirm target-site based cross-resistance, despite the fact that the three mite growth inhibitors belong to chemically diverse classes^87–89^. In a recent study, Douris, *et al*.^20^ found a mutation (I1042M) in CHS1 gene of *Plutella xylostella* resistant to benzoylureas (BPUs), at the position corresponding to I1017F in *T. urticae*. Using CRISPR/Cas9 approach coupled with homology directed repair (HDR), both the lepidopteran and spider mite mutations (I1056M/F) were introduced in the *Drosophila CHS1* gene (kkv). Flies carrying either of two mutations were found highly resistant to etoxazole, but also to a number of BPUs and the hemipteran chitin biosynthesis inhibitor buprofezin. The study, together with the results reported here, provide convincing evidence that chitin synthesis inhibitors BPUs, buprofezin and mite growth inhibitors, etoxazole, hexythiazox and clofentezin all directly interact with CHS1 and share a similar molecular MoA.

## Conclusions

Resistance mechanisms in insects and mites can be complex and the relative strength of target-site mutations in resistance phenotypes is often not well known. Here, we have used a marker-assisted backcrossing approach to look at the phenotypic effect of the main and currently relevant target-site mutations reported to confer resistance to abamectin, pyrethroids, mite growth inhibitors and QoI. Mutations in VGSC, CHS and cytb confer high levels of resistance and their presence in populations alone is enough to cause field failure after acaricide treatment. In contrast, although we confirmed the functional importance of GluCl mutations and the cumulative effect of mutations in multiple channels, mutations in only two channels genes does not lead to the high resistance levels that have been reported for abamectin resistance. Overall, our results functionally validate the importance of mutations that have been inferred from correlation analysis and genetic mapping.

## Electronic supplementary material

Supplementary Tables
