# Supplementary material for: The relative contribution of target-site mutations in complex acaricide resistant phenotypes as assessed by marker assisted backcrossing in Tetranychus urticae
Source: Sci Rep. 2017 Aug 23;7:9202. doi: 10.1038/s41598-017-09054-y (PMC5569037; doi:10.1038/s41598-017-09054-y)
Supplement: Supplementary file 1 — Supplementary Tables [file 41598_2017_9054_MOESM1_ESM.pdf]

The relative contribution of target-site mutations in complex acaricide resistant phenotypes as assessed by marker assisted backcrossing in *Tetranychus urticae*.

Maria Riga, Sabina Bajda, Christos Themistokleous, Stavrini Papadaki, Maria Palzewicz, Wannes Dermauw, John Vontas, Thomas Van Leeuwen

Table S1: Primers and probes used in this study

Name	*Sequence (5'-3')*	*Description*	*Reference*
G314D_F (Primer)	CACGTCAAATATCAGGAATCAATGCAT	GluCl1 mutation (G314D)	[1,2]
G314D_R (Primer)	GGCAAATTCAATGAGAGCACCAAAA		
G314D_VIC (Probe)	TTGACATTTGGACAGATTG		
G314D_FAM (Probe)	TGACATTTGGACAGGTTG		
Tu_GluCl1_diag_F	TTGGATTGACCCTAACTCAGCA		
Tu_GluCl1_diag_R	TTGCACCAACAATTCCTTGA		
G326E_F (Primer)	TCCACCGGTCAGTTACATTAAAGC	GluCl3 mutation (G326E)	
G326E_R (Primer)	CAAACTCTAGGAGGGCACCAAAA		
G326E_VIC (Probe)	TTGGACCGAAGTCTG		
G326E_FAM (Probe)	TTTGGACCGGAGTCTG		
Tu_GluCl3_diag_F	CCGGGTCAGTCTTGGTGTTA		
Tu_GluCl3_diag_R	CACCACCAAGAACCTGTTGA		
F1538I_F (Primer)	AACAACCAGTTTATGAAAATAGTATTCTGATGTACTTA	VGSC mutation (F1538I)	[2,3]
F1538I_R (Primer)	CACCTCCTTTCTTTTTTTGTTCATTAAAATTATCAATAATG		
F1538I_VIC (Probe)	TTTTTGGCTCTTTTATCACAC		
F1538I_ FAM (Probe)	TTTTGGCTCTTTTTTCACAC		
kdrF4	CAACATTCAAAGGTTGGACAAT		
kdr R1	TCTTCCGTCATCAACATCTCC		
kdrF5	TGATTGTTTTCCGTGTCCTG	VGSC mutation (L1024V)	
kdrR5	CTGCGAAGCTGCTTAAGTCC		
kdrF2	TGCATCTCAATTGTCCAAGG	VGSC mutation (A1215D)	
kdrR2	GTTTCTTCCAGGCAACATGG		
PEWY_F	AAAGGCTCATCTAACCAAATAGG	Cytb mutation (P262T)	[4]
PEWY_R	AATGAAATTTCTGTAAAAGGGTATTC		
cytbWT_F	CGGAATAATTTTACAAATAACTCATGC	Cytb mutation (G126S, S141F)	
cytbWT_R	TGGTACAGATCGTAAAATTGCG		
TuCHS1_F	CTTCACCGTCTGCCGTATTT	Chitin synthase mutation (I1017F)	[5]
TuCHS1_R	CTTTTCGTCGTTTGGTTTGG		

Table S2: Toxicity of abamectin and milbemectin to adult females of backcrossed lines GluCl1_C (G314/G314), GluCl1_R1-3 (G314D/G314D), GluCl3_C (G326/G326), GluCl3_R1-3 (G326E/G326E), GluCl1+3_C (G314/G314; G326/G326), GluCl1+3_R1-R3 (G314D/G314D; G326E/G326E) and their parental strain (Wasatch, MAR-AB). [a] Number of the mites used in toxicity tests. [b] Resistance ratio compared to Wasatch. a: Treatment effect was significantly different when compared to Wasatch

Compound	***Strain***	***Genotype***	N^a	LC_{50} *mg* l^{-1} *(95% CL)*	***Slope (±SE)***	χ^2 *(df)*	*RR (95% CL)*[b]
Abamectin	Wasatch	G314;G326	545	0.4 (0.3 ; 0.4)	5.0 (± 0.6)	9 (16)	-
	MAR-AB	G314D;G326E	425	512.2 (430.8 ; 578.7)a	4.3 (± 0.8)	14 (16)	1,354.9 (1,147.9 ; 1,599.3)
	GluCl1_C	G314	370	0.3 (0.3 ; 0.4)	4.3 (± 1.0)	11 (13)	0.9 (0.8 ;1.1)
	GluCl3_C	G326	474	0.4 (0.3 ; 0.4)	6.5 (± 1.3)	18 (16)	1.0 (0.9 ; 1.2)
	GluCl1+3_C	G314;G326	659	0.4 (0.4 ; 0.5)	4.1 (± 0.6)	22 (16)	1.1 (0.9 ; 1.3)
	GluCl1_R1	G314D	555	0.7 (0.7 ; 0.8)a	5.5 (±1.0)	21 (19)	1.9 (1.7 ; 2.2)
	GluCl1_R2	G314D	394	0.6 (0.6 ; 0.7)a	6.9 (± 0.9)	15 (13)	1.7 (1.5 ; 1.9)
	GluCl1_R3	G314D	447	1.1 (1.0 ; 1.2)a	6.5 (± 1.1)	10 (13)	2.9 (2.5 ; 3.3)
	GluCl3_R1	G326E	519	1.3 (1.1 ; 1.4)a	6.8 (± 0.8)	9 (16)	3.3 (2.9 ; 3.8)
	GluCl3_R2	G326E	466	1.3 (1.1 ; 1.5)a	4.7 (±0.6)	15 (16)	3.4 (3.0 ; 4.0)
	GluCl3_R3	G326E	502	1.1 (1.0 ; 1.2)a	5.9 (± 0.7)	8 (16)	2.9 (2.5 ; 3.3)
	GluCl1+3_R1	G314D;G326E	513	7.5 (6.4 ; 8.5)a	3.7 (± 0.4)	8 (16)	19.8 (16.8 ; 23.3)
	GluCl1+3_R2	G314D;G326E	399	3.8 (3.3 ; 4.3)a	5.7 (± 0.8)	11 (13)	10.1 (8.7 ; 11.7)
	GluCl1+3_R3	G314D;G326E	396	3.6 (3.1 ; 4.0)a	5.1 (± 0.7)	8 (13)	9.5 (8.1 ; 11.1)
Milbemectin	Wasatch	G314;G326	416	0.9 (0.6 ; 1.1)	5.5 (± 1.2)	15 (13)	-
	MAR-AB	G314D;G326E	409	65.4 (52.1 ; 76.4)a	3.9 (± 0.6)	14 (12)	71.7 (55.9 ; 92.0)
	GluCl1_C	G314	448	0.9 (0.8 ; 1.0)	7.5 (± 1.0)	7 (13)	1.0 (0.8 ; 1.3)
	GluCl3_C	G326	436	0.7 (0.6 ; 0.8)a	7.4 (± 1.3)	17 (16)	0.8 (0.6 ; 0.9)
	GluCl1+3_C	G314;G326	417	0.8 (0.7 ; 1.0)	6.4 (± 1.0)	18 (13)	0.9 (0.7 ; 1.2)
	GluCl1_R1	G314D	479	1.5 (1.3 ; 1.6)a	6.8 (± 1.5)	13 (16)	1.6 (1.3 ; 2.0)
	GluCl1_R2	G314D	444	1.3 (1.1 ; 1.4)a	6.2 (± 1.1)	18 (16)	1.4 (1.1 ; 1.7)
	GluCl1_R3	G314D	452	1.4 (1.3 ; 1.6)a	4.1 (± 0.7)	7 (16)	1.6 (1.2 ; 2.0)
	GluCl3_R1	G326E	532	1.4 (1.2 ; 1.7)a	2.6 (± 0.4)	10 (16)	1.6 (1.2 ; 2.0)
	GluCl3_R2	G326E	388	1.3 (1.0 ; 1.5)a	3.2 (± 0.6)	21 (16)	1.4 (1.1 ; 1.8)
	GluCl3_R3	G326E	431	1.4 (1.1 ; 1.7)a	2.8 (± 0.5)	9 (16)	1.5 (1.1 ;2.0)
	GluCl1+3_R1	G314D;G326E	360	7.0 (5.3 ; 9.1)a	1.9 (± 0.2)	17 (13)	7.7 (5.7 ; 10.3)
	GluCl1+3_R2	G314D;G326E	472	12.6 (10.1 ; 15.1)a	2.6 (± 0.3)	12 (16)	13.7 (10.3 ; 18.2)
	GluCl1+3_R3	G314D;G326E	517	11.4 (9.7 ; 13.4)a	3.5 (± 0.4)	15 (16)	12.5 (9.7 ; 16.1)

Table S3. Toxicity of bifenazate to adult females of back-crossed lines Cytb_R4-5 (G126S+ S141F/G126S+S141F) and Cytb_R1-R3 (P262T/P262T) and toxicity of acequinocyl to Cytb_R4-5, supplemented with toxicity data for both acaricides versus parental resistant strain BR-VL and bifenazate versus parental resistant strain HOL3. [a] Number of the mites used in toxicity tests. [b] Resistance ratio compared to Wasatch. a: Treatment effect was significantly different when compared to Wasatch

Compound	*Strain*	*Genotype*	*N*[a]	*LC_{50} mg l^{-1} (95%CL)*	*Slope (± SE)*	*χ^2 (df)*	*RR (95%CL)*[b]
Bifenazate	Wasatch	G126+S141, P262	873	2.4 (1.7; 3.1)	2.7 (± 0.2)	107.7 (22)	-
	HOL3	P262T	178	>5,000a	-	-	>2,000
	BR-VL	G126S+S141F	873	>5,000a	-	-	>2,000
	Cytb_R1	P262T	783	960.0 (821.5; 1,092.4)a	2.51 (± 0.2)	17.1 (18)	400.4 (332.5; 482.2)
	Cytb _R2	P262T	533	1,400.6 (1,118.4; 1,686.9)a	2.56 (± 0.2)	35.2 (18)	584.1 (488.0; 699.2)
	Cytb _R3	P262T	655	886.1 (444.4; 1,543.1)a	1.58 (± 0.2)	159.4 (21)	369.6 (297.5; 459.1)
	Cytb_R4	G126S+S141F	278	>5,000a	-	-	>2,000
	Cytb_R5	G126S+S141F	320	>5,000a	-	-	>2,000
Acequinocyl	Wasatch	S141	603	7.6 (6.5; 8.6)	4.81 (± 0.4)	30.7 (18)	-
	BR-VL	G126S+S141F	708	218.5 (172.3–265.9)a	2.31 (± 0.2)	40.6 (22)	28.9 (24.2; 34.6)
	Cytb_R4	G126S+S141F	1,346	205.9 (180.2; 231.4)a	3.38 (± 0.3)	36.3 (30)	27.3 (23.6; 31.6)
	Cytb_R5	G126S+S141F	677	302.7 (233.9; 370.7)a	2.74 (± 0.3)	33.8 (18)	40.1 (33.4; 48.1)

Table S4**:** Toxicity of etoxazole, hexythiazox and clofentezine to larvae of *T. urticae* of back-crossed lines CHS1_C (I1017/I1017 genotype), CHS1_R1-R3 (I1017F/I1017F genotype), and their parental strains (Wasatch and EtoxR). [a] Number of larvae used in toxicity tests with etoxazole and hexythiazox or number of eggs used in toxicity tests with clofentezine. [b] Resistance ratio compared to Wasatch. a: Treatment effect was significantly different when compared to Wasatch

Compound	***Strain***	***Genotype***	***N[a]***	***LC_{50} mg l^{-1} (95%CL)***	***Slope (± SE)***	***χ^2 (df)***	***RR (95%CL)[b]***
Etoxazole	Wasatch	I1017	1,143	0.1 (0.08; 0.1)	3.3 (± 0.3)	74.9 (18)	-
	EtoxR	I1017F	180	>5,000a	-	-	>40,000
	CHS1_C	I1017	510	0.1 (0.07; 0.12)a	2.3 (± 0.3)	15.9 (10)	0.8 (0.7; 1.0)
	CHS1_R1	I1017F	920	>5,000a	-	-	>40,000
	CHS1_R2	I1017F	530	>5,000a	-	-	>40,000
	CHS1_R3	I1017F	579	>5,000a	-	-	>40,000
Hexythiazox	Wasatch	II1017	953	1.1 (0.9; 1.4)	3.8 (± 0.3)	55.8 (13)	-
	EtoxR	I1017F	245	>5,000a	-	-	>4,000
	CHS1_C	I1017	947	1.9 (1.3; 2.5)a	2.6 (± 0.2)	101.0 (18)	1.7 (1.4; 1.9)
	CHS1_R1	I1017F	422	>5,000a	-	-	>4,000
	CHS1_R2	I1017F	531	>5,000a	-	-	>4,000
	CHS1_R3	I1017F	472	>5,000a	-	-	>4,000
Clofentezine	Wasatch	II1017	1,177	2.3 (1.8; 2.9)	2.9 (± 0.2)	86.0 (18)	-
	EtoxR	I1017F	305	>5,000a	-	-	>2,000
	CHS1_C	I1017	1,037	1.6 (1.3; 2.1)a	2.9 (± 0.2)	65.8 (16)	0.7 (0.6; 0.8)
	CHS1_R1	I1017F	598	>5,000a	-	-	>2,000
	CHS1_R2	I1017F	558	>5,000a	-	-	>2,000
	CHS1_R3	I1017F	700	>5,000a	-	-	>2,000

References

1 Dermauw, W. *et al.* The cys-loop ligand-gated ion channel gene family of *Tetranychus urticae:* Implications for acaricide toxicology and a novel mutation associated with abamectin resistance. *Insect Biochem Molec.* **42**, 455-465 (2012).

2 Ilias, A., Vassiliou, V. A., Vontas, J. & Tsagkarakou, A. Molecular diagnostics for detecting pyrethroid and abamectin resistance mutations in *Tetranychus urticae*. *Pest Biochem Physiol.* **135**, 9-14 (2017).

3 Khajehali, J., Van Nieuwenhuyse, P., Demaeght, P., Tirry, L. & Van Leeuwen, T. Acaricide resistance and resistance mechanisms in *Tetranychus urticae* populations from rose greenhouses in the Netherlands. *Pest Manag Sci.* **67**, 1424-1433 (2011).

4 Van Leeuwen, T. *et al.* Mitochondrial heteroplasmy and the evolution of insecticide resistance: Non-Mendelian inheritance in action. *PNAS.* **105**, 5980-5985 (2008).

5 Demaeght, P. *et al.* High resolution genetic mapping uncovers chitin synthase-1 as the target-site of the structurally diverse mite growth inhibitors clofentezine, hexythiazox and etoxazole in *Tetranychus urticae*. *Insect Biochem Mol Biol.* **51**, 52-61 (2014).
